# Gemcitabine combination therapies induce apoptosis in uterine carcinosarcoma patient-derived organoids

**DOI:** 10.3389/fonc.2024.1368592

**Published:** 2024-03-13

**Authors:** Matías J. Dahl, Kristopher A. Lofgren, Cleo Haugen, Gil E. Harmon, Sarah P. Hughes, Karen D. Cowden Dahl

**Affiliations:** ^1^ Kabara Cancer Research Institute, Gundersen Medical Foundation, La Crosse, WI, United States; ^2^ Department of Hematology and Oncology, Gundersen Health Systems, La Crosse, WI, United States; ^3^ Department of Pathology, Gundersen Health Systems, La Crosse, WI, United States

**Keywords:** uterine carcinosarcoma, patient-derived organoids, chemotherapy, apoptosis, carboplatin, paclitaxel, gemcitabine

## Abstract

Uterine carcinosarcoma (UCS) is a rare but aggressive endometrial cancer. Survival outcomes for women diagnosed with UCS remain poor with lower survival than those of endometrioid or high-grade serous uterine cancers. The histopathological hallmark of carcinosarcoma is the presence of both sarcomatous and carcinomatous elements. The survival rates for UCS have not improved for over 40 years; therefore, there is a profound need to identify new treatments. To investigate novel chemotherapy treatment combinations for UCS, we generated a UCS patient-derived organoid (PDO) cell line from a patient that received neoadjuvant treatment with paclitaxel and carboplatin. The PDO cell line (UCS1) was grown in three-dimensional domes. The PDO domes were treated with six individual chemotherapies or nine combinations of those six drugs. Cell death in response to chemotherapy was assessed. We found that the six monotherapies had minimal effectiveness at inducing cell death after 48 h of treatment. The combination of paclitaxel and carboplatin (which is the standard-of-care chemotherapy treatment for UCS) led to a small increase in apoptosis compared with the monotherapies. Importantly, when either carboplatin or paclitaxel was combined with gemcitabine, there was an appreciable increase in cell death. In conclusion, for the UCS1 patient-derived tumor cells, gemcitabine combinations were more effective than carboplatin/paclitaxel. Our data support the use of PDOs to predict responses to second-line chemotherapy.

## 1 Introduction

High-grade endometrial tumors are composed of FIGO grade 3 endometrioid, serous, clear cell, undifferentiated/dedifferentiated carcinomas and carcinosarcomas (1). While uterine serous carcinoma is rare compared with endometrioid carcinoma, patients with serous carcinomas have a higher incidence of recurrence and poorer prognosis (2). Uterine carcinosarcoma (UCS) is a high-grade and highly aggressive cancer, which represents roughly 5% of all endometrial malignancies. Survival outcomes for women diagnosed with UCS remain poor and worse than that of endometrioid or high-grade serous histologies (3). The median survival of patients with UCS is less than 2 years and the overall survival rate ~33% (4). Carcinosarcomas are characterized by the presence of both sarcomatous and carcinomatous elements. The carcinomatous component can range from low to high grade with the majority being high grade. The sarcomatous component is further characterized as being either homologous or heterologous. The homologous sarcoma has evidence of high-grade undifferentiated round or spindle-cell proliferation with features resembling leiomyosarcoma, endometrial stromal sarcoma, or fibrosarcoma. The heterologous sarcoma, which accounts for around half of the cases, can have histophenotypes resembling cartilaginous, rhabdosarcomatous, chondrosarcomatous, or osteosarcomatous differentiation (5). The percentage of carcinoma and sarcoma varies from patient to patient (6). The component invading the myometrium or metastatic in the lymph nodes is often the carcinomatous component. However, metastases can include both the carcinomatous and sarcomatous components or only the sarcomatous element, although this is less frequent (7). There are a number of factors in UCS that correlate with poorer prognosis. Some of these factors include lymph node metastasis, heterologous histopathology, higher than 25% stromal composition, elevated expression of vascular endothelial growth factor, and increased microvessel density (8–10). Therefore, due to the complicated nature and heterogeneity of UCS, responses to chemotherapy are currently difficult to predict.

The incidence of UCS is increasing in the USA (4). Optimal treatment for UCS is debated. Given that UCS is rare, it is a challenging tumor to investigate potential treatment options. Additionally, few clinical trials are applicable for UCS patients. When compared with uterine adenocarcinomas, UCS presents with a higher grade and frequency of metastasis, resulting in lower overall survival (4). The presence of two tumor histologies in UCS makes treatment and cure challenging. Importantly, the ratio of carcinoma to sarcoma impacts survival as sarcoma dominance (SD) (>50% of the tumor is sarcomatous) correlates with poorer prognosis (4). Most UCS patients receive carboplatin and paclitaxel with or without radiation following a debulking surgery. Surgery usually entails a hysterectomy with bilateral salpingo-oophorectomy and lymphadenectomy for staging. The response to chemotherapy in UCS depends on the grade of tumor, type of sarcoma, and if there is SD (11). The clinical data suggest that carcinoma and sarcoma tumor cells respond differently to therapy and implies that targeting the sarcoma tumor cells separately from the carcinoma may improve chemotherapy response rates. Currently, the median response rates for UCS to first- and second-line chemotherapy are 37.5% and 5.5%, respectively (4). Therefore, there is a crucial need to enhance our understanding about UCS progression in order to develop more effective therapies and improve survival.

Genetic evidence suggests that UCS is epithelial derived and that the sarcoma component arises from dedifferentiation of the epithelial/carcinoma precursor (12, 13). How UCS cells are programmed to become both carcinoma and sarcoma cells is unknown. Genetic analysis revealed that sarcoma and carcinoma components of UCS share the same driver mutations including TP53, PIK3CA FBXW7, PTEN, and KRAS (14). It is hypothesized that epithelial to mesenchymal transition (EMT) signals induce differentiation of the sarcomatous portion of the UCS. In particular, the sarcoma tumor cells have a higher EMT score (expression EMT markers) than the carcinoma cells (14–16). SD tumors tend to have a heterologous histology and express EMT markers (11, 17). Factors that may contribute to UCS EMT are repression of the miR-200 family of microRNAs (miRNAs) and TGFβ (18–20). Overexpression of miR-200 in UCS xenografts induced epithelial morphology and decreased tumor growth (20). TGFβ promotes EMT in UCS cells, and inhibition of TGFβ reduces viability and differentiation of UCS cells (18, 19). In support of this, TGFβ is demonstrated to promote EMT and a cancer stem cell (CSC) phenotype (21, 22). Additionally, adipocytes have the ability to induce EMT in cancer cells (23–25). These studies suggested that the UCS tumors exhibit plasticity and the tumor microenvironment (TME) influences UCS tumor differentiation.

Patient-derived organoid (PDO) cell lines have been generated for endometrial cancers including UCS (26). PDOs are tumor cells that are harvested directly from a fresh patient tumor or tumor cells such as ascites and cultured in 3D. By culturing the cells in a 3D matrix, the cells retain some of the structure and behavior as found in the original tumor and exhibit more accurate responses to treatments than established tumor cell lines. PDOs also enable us to study the physical properties of tumor cells and response to therapy of individual tumors *ex vivo (* 27). These PDOs have the potential to determine chemotherapy sensitivities for first-line chemotherapy and subsequent rounds of chemotherapy (28). The goal of our study was to examine the impact of different chemotherapy treatments for UCS on a primary patient-derived organoid (PDO) line. The PDO line was generated from a primary tumor of a patient with UCS who had received neoadjuvant carboplatin and paclitaxel. We treated organoids with six different chemotherapy drugs and nine combinations of those drugs. We chose to use carboplatin and paclitaxel in our studies because this combination is the standard of care for UCS. Additionally, we used ifosfamide, docetaxel, doxorubicin, and gemcitabine since these drugs either alone or in combination are possible therapies for uterine sarcoma. According to the NCCN guidelines, doxorubicin is preferred; however, gemcitabine, ifosfamide, and docetaxel/gemcitabine can be used for sarcoma treatment. We assessed how the treatments impacted cell morphology and survival.

## 2 Results

### 2.1 Derivation of patient-derived organoid cell line UCS1

**Figure 1:**
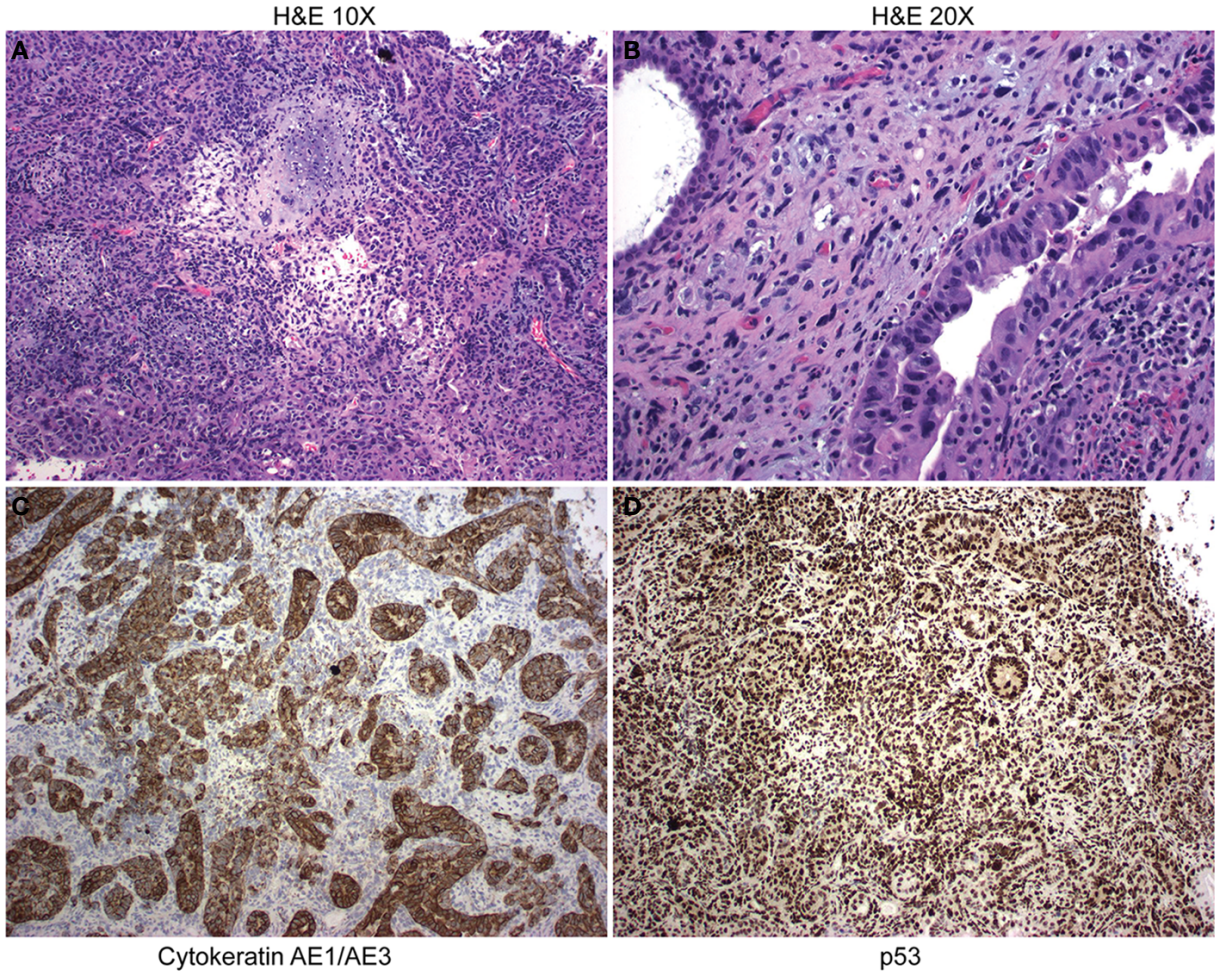
Pathology from uterine carcinosarcoma (UCS) patient tumor. **(A)** H&E staining at 10× demonstrates UCS tumor with serous carcinoma and chondroid sarcomatous differentiation. **(B)** H&E at 20× magnification. **(C)** Immunohistochemistry demonstrates staining for cytokeratin. **(D)** Immunohistochemistry demonstrates nuclear accumulation of p53.

A patient with UCS was selected for the study. This patient had stage IVB at the time of diagnosis. The carcinoma component of the tumor was poorly differentiated serous carcinoma, and the sarcomatous portion was undifferentiated with a small amount of chondroid differentiation. The initial biopsy contained 95% carcinoma and 5% sarcoma. H&E staining demonstrated the UCS diagnosis (**Figures 1A, B**). Immunohistochemistry (IHC) cytokeratin AE1AE3 demonstrated cytokeratin in the carcinomatous portion of the tumor, whereas the sarcomatous portion was negative for cytokeratin (**Figure 1C**). IHC staining on the tumor found diffuse strong p53 positivity in both carcinomatous and sarcomatous tumor cells (**Figure 1D**). Tumor was also focally positive vimentin (not shown). Tempus sequencing on tumor biopsy found a loss of function mutation in TP53 at A159V. The patient was treated with three rounds of neoadjuvant carboplatin and paclitaxel. After the chemotherapy regime, the patient underwent a total abdominal hysterectomy with bilateral salpingo-oophorectomy, omentectomy, and tumor debulking. Response to therapy was CRS2 (moderate). At the time of surgery, a tumor specimen was collected and processed into a single-cell suspension. The single cells were suspended in a three-dimensional (3D) dome composed of an artificial basement membrane to generate a UCS organoid cell line (UCS1).

### 2.2 Characterization of UCS1

**Figure 2:**
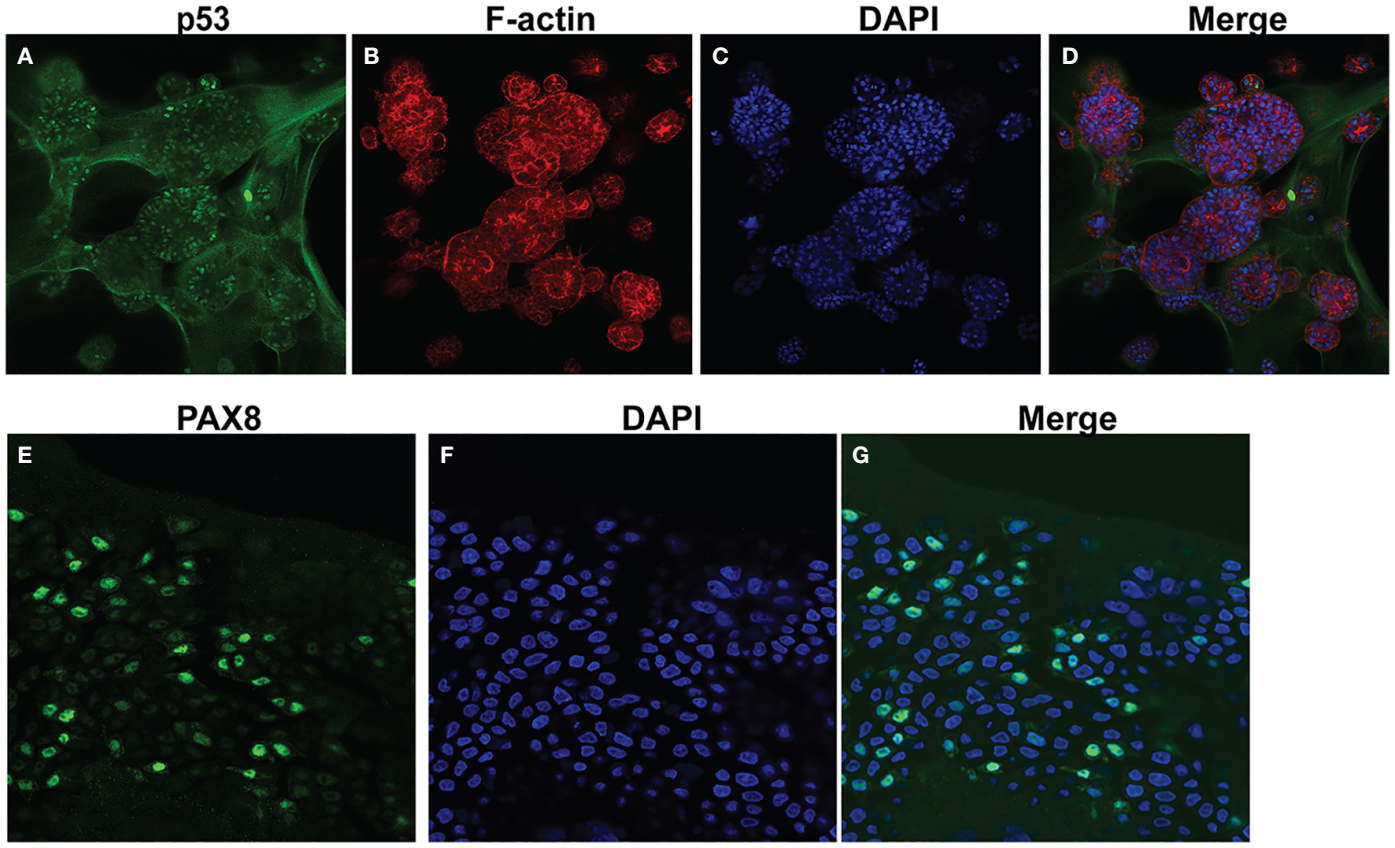
Characterization of UCS1 organoids by immunofluorescence and confocal microscopy. **(A–D)** Depicts staining for p53 **(A)**, Texas-Red phalloidin (F-actin) **(B)**, and DAPI **(C)** on UCS PDOs (10x magnification). **(D)** is the merged image of **(A–C)**. **(E–G)** Demonstrates nuclear expression of PAX8 in organoids (63× magnification).

**Figure 3:**
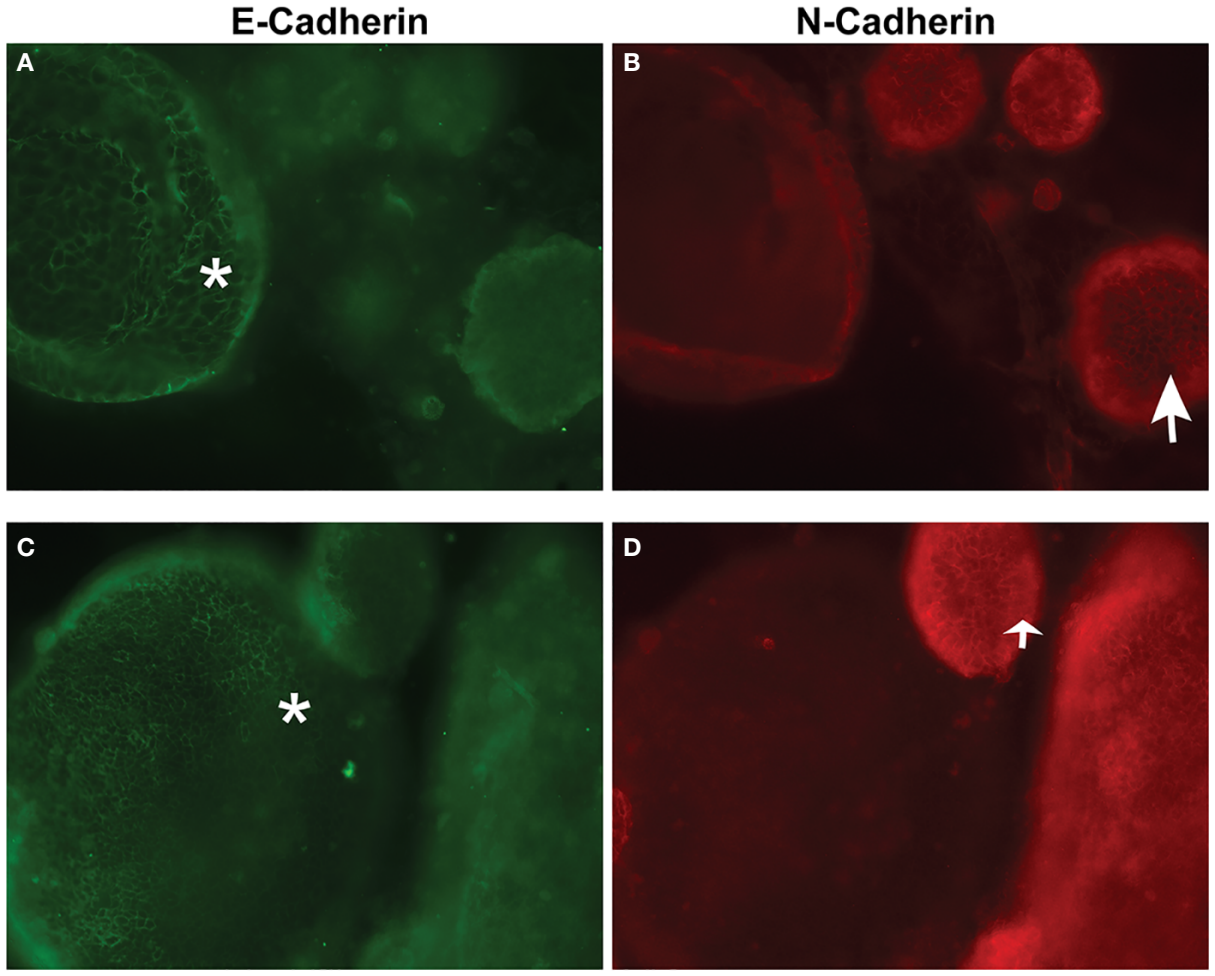
Immunofluorescence and confocal microscopy for E-cadherin and N-cadherin in UCS1 organoids. **(A, C)** are staining for E-cadherin, and **(B, D)** are staining for N-cadherin. **(A, B)** are different fields of cells than **(C, D)**. *: E-cadherin-positive cells; arrow: N-cadherin positive cells (63× magnification).

**Figure 4:**
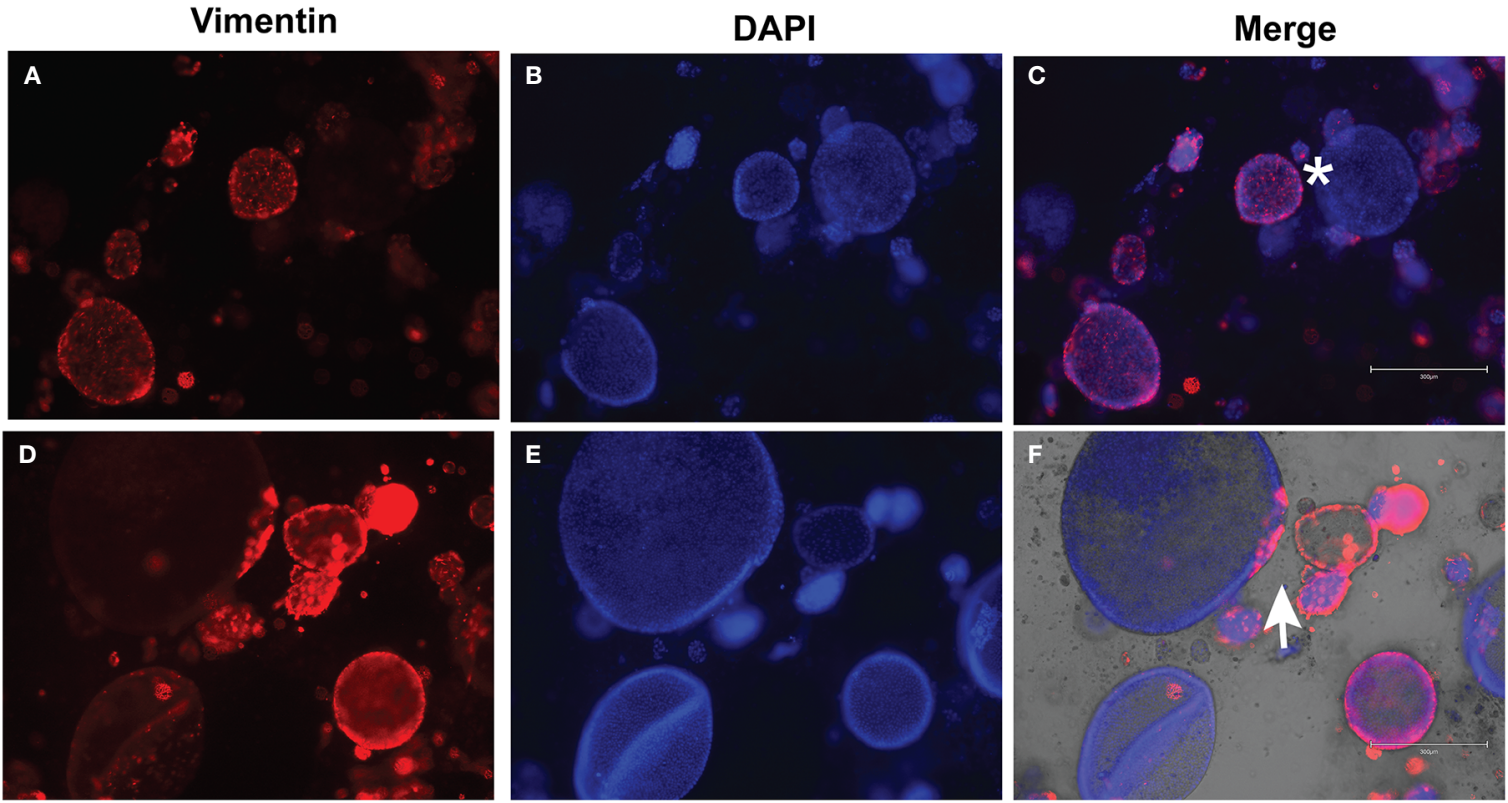
Immunofluorescence for vimentin on UCS1 organoids. UCS1 organoids were stained for vimentin **(A, D)** and DAPI **(B, E)**. **(A–C)** and **(D–F)** are two independent fields of spheroids. **(C, F)** are merged images. *: vimentin-negative cells; arrow: vimentin-positive cells (20× magnification).

**Figure 5:**
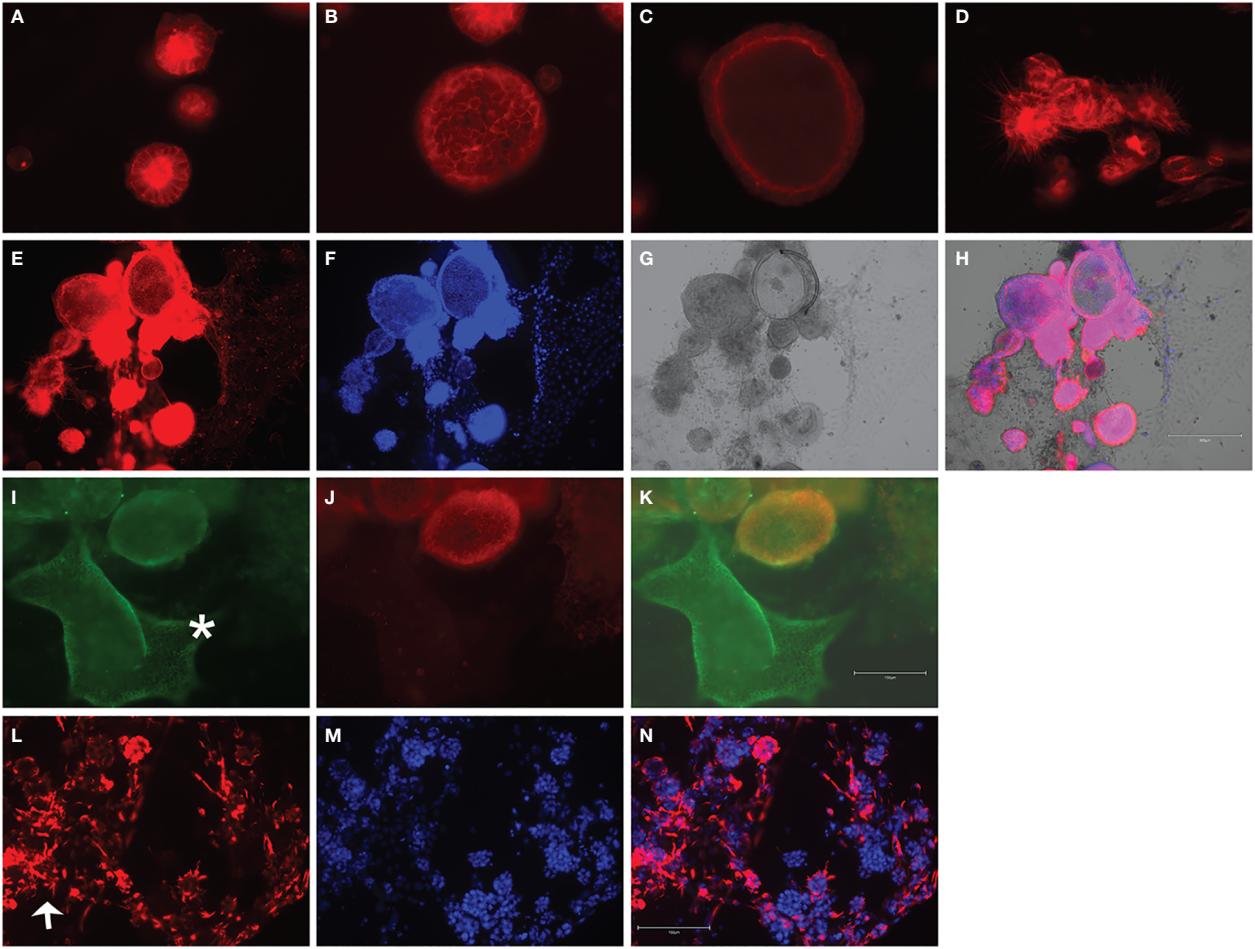
Fluorescent staining of UCS1 organoids demonstrates diverse morphologies. F-Actin staining in **(A–D)** denotes types of cellular organization observed in UCS1 cultures. Panels **(E–H)** demonstrate through F-actin staining in **(E)**, DAPI **(F)**, and brightfield **(G)** that tumor cells can differentiate and migrate/extend from spheroids. H is the merged image of **(E–G)**. Panels **(I–K)** demonstrate that migratory (non-spheroid) tumor cells can express E-cadherin (green staining in **I**, *E-cadherin-positive cells). **(J)** is N-cadherin, and **(K)** is a merge of **(I)** and **(J)**. Panel **(L)** is vimentin staining on non-spheroid tumor cells, and **(N)** is the merged image with DAPI in **(M)**. Arrow = vimentin-positive cells. 20× magnification.

Following development of the UCS1 line, we conducted further characterization using immunofluorescence (IF) and fluorescent staining on UCS1 organoids to assess the expression or localization/organization of F-actin, p53, Pax8, vimentin, E-cadherin, and N-cadherin. Consistent with the pathology findings on the primary tumor, focal expression of p53 (**Figure 2A**) was identified in organoids. Texas Red phalloidin staining and DAPI staining demonstrate F-actin organization and nuclei in organoids (**Figures 2B–D**). Organoids also contained PAX8-positive cells, demonstrating the Mullerian origin of the cells (**Figures 2E–G**). Our next goal was to examine the morphology of the spheroids (referring to isolated 3D spheres of PDO cells) generated from UCS1 and determine if they exhibited epithelial and/or mesenchymal markers. IF for E-cadherin, N-cadherin, and vimentin was conducted. We found that individual UCS1 spheroids typically expressed either E-cadherin (**Figures 3A, C**) or N-cadherin (**Figures 3B, D**). Some individual live UCS1 spheroids expressed vimentin (**Figures 4A, B**) while others did not express vimentin (**Figures 4B, C**). Mixed morphology did occur in some organoid structures, but it was rare (**Figures 4D–F**). In our characterization of the organoids, we found that UCS1 cells formed spheroids with a variety of morphologies (**Figures 5A–D**). **Figure 5** demonstrates the diversity in organoid organization. Texas Red phalloidin staining for F-actin demonstrated that some clusters had apical columnar organization (**Figure 5A**). Other spheroids were entirely cobblestone, exhibited a ring of cells on the surface of the sphere, or were unorganized (**Figures 5B–D**). On occasion, we had tumor cells migrate out from spheres (**Figures 5E–N**). These non-spheroid/differentiated cells could be epithelial as indicated through expression of E-cadherin (**Figures 5I–K**) or mesenchymal as indicated by vimentin expression (**Figures 5L–N**). Our characterization of the organoid morphology demonstrated that like UCS tumors, the organoids had diverse morphology and expression of epithelial and mesenchymal markers.

### 2.3 UCS1 organoid response to therapy

Due to the aggressive nature and poor survival associated with UCS, we hypothesized that UCS-derived PDOs may be used to identify alternate options for chemotherapy. We investigated the impacts of individual and combination chemotherapy treatments on the UCS1 line. We treated the UCS1 organoids with six different chemotherapy drugs and nine combinations of those drugs (**Tables 1**, **2**).

**Table 1:** Doses of chemotherapy used in treating UCS1 organoids.

Drug	Dose 1	Dose 2	Dose 3	Dose 4	Dose 5
**Carboplatin**	Vehicle	25 nM	50 nM	**100 nM**	500 nM
**Docetaxel**	Vehicle	10 nM	50 nM	**100 nM**	10 μM
**Doxorubicin**	Vehicle	10 nM	100 nM	**1 μM**	50 μM
**Gemcitabine**	Vehicle	100 nM	1 μM	**10 μM**	100 μM
**Ifosfamide**	Vehicle	–	250 μM	**500 μM**	1mM
**Paclitaxel**	Vehicle	5 nM	50 nM	**500 nM**	5 μM

**Table 2:** Chemotherapy combinations used for treating UCS1 organoids.

Combination number	Drug combination
Combo 1: (C1)	100 nM carboplatin 500 nM paclitaxel
Combo 2: (C2)	100 nM carboplatin 100 nM docetaxel
Combo 3: (C3)	100 nM carboplatin 10 μM gemcitabine
Combo 4: (C4)	100 nM carboplatin 500 μM ifosfamide
Combo 5: (C5)	500 μM ifosfamide 500 nM paclitaxel
Combo 6: (C6)	100 nM doxorubicin 500 nM paclitaxel
Combo 7: (C7)	10 μM gemcitabine 500 nM paclitaxel
Combo 8: (C8)	100 nM doxorubicin 10 μM gemcitabine
Combo 9: (C9)	100 nM doxorubicin 100 nM docetaxel

**Figure 6:**
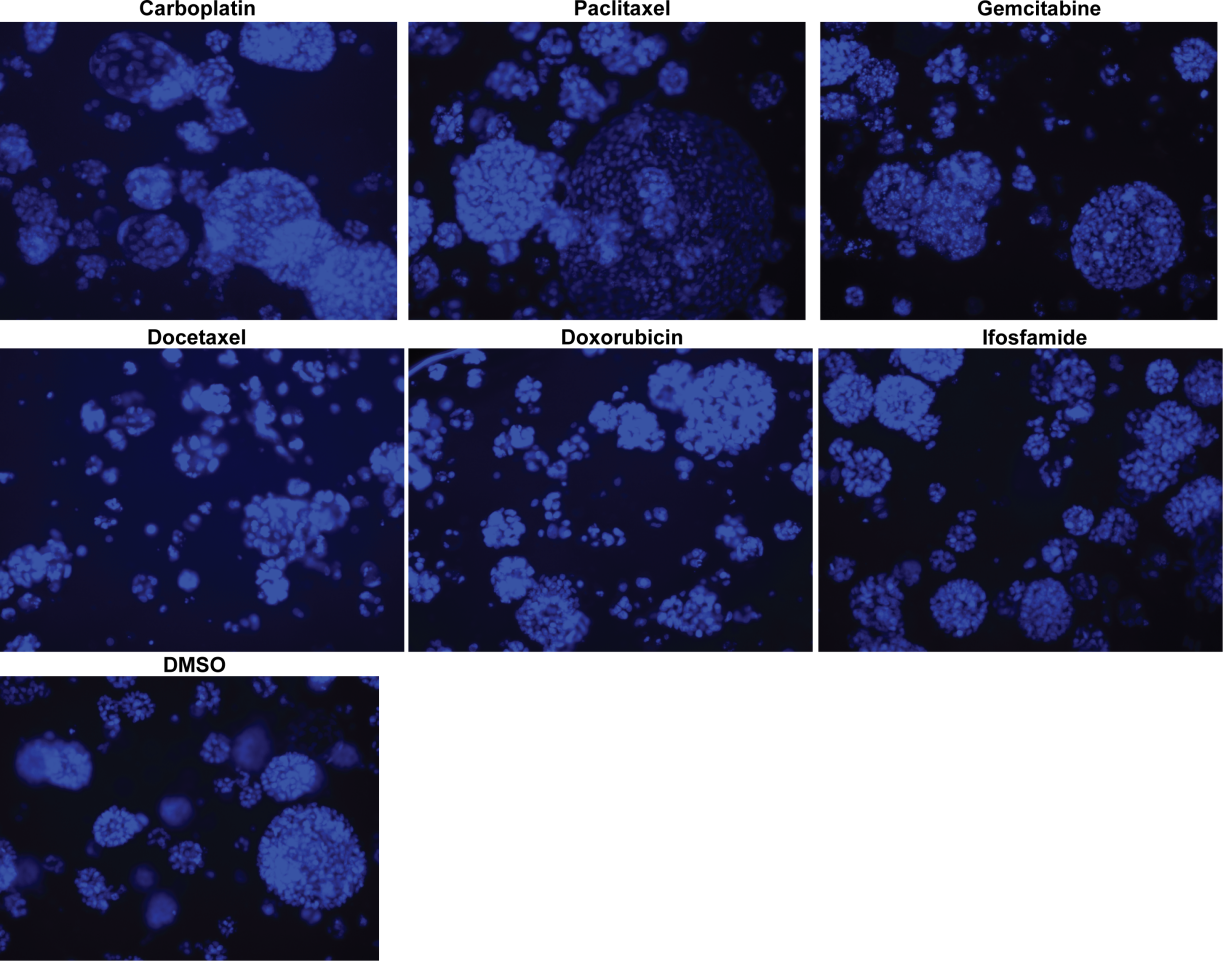
Monotherapy treatments of UCS1 organoids did not significantly alter nuclear integrity. DAPI staining was conducted on UCS1 cells treated with monotherapies listed in **Table 1** for 48 h.

**Figure 7:**
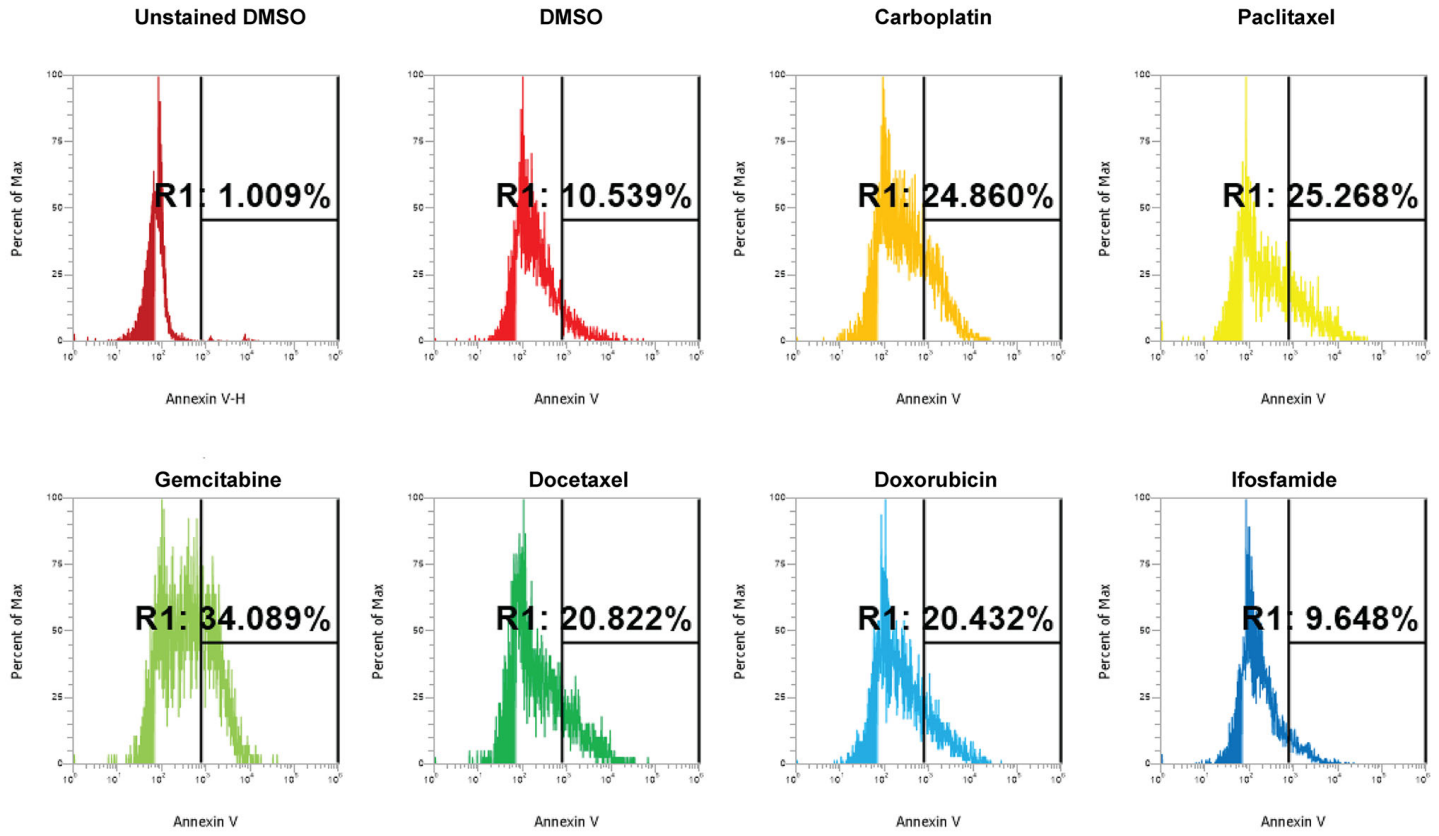
Induction of apoptosis by individual chemotherapy treatments in UCS1 organoids. UCS1 cells were treated for 48 h with DMSO, carboplatin, paclitaxel, gemcitabine, docetaxel, doxorubicin, or ifosfamide (at indicated doses in **Table 1**). Annexin V staining followed by flow cytometry was conducted. Percentages of Annexin V-positive cells are indicated as compared with unstained and untreated UCS1 cultures.

We conducted dose curve analysis on organoids to identify appropriate ranges of drugs to be used in further experiments (**Supplementary Figure 1**). We treated UCS1 organoids with four doses of each of the six drugs (**Table 1**). We chose dose 4 (Bold concentrations in **Table 1**) for each of the individual therapies for further studies due to activation of CellEvent 3/7 and Annexin V staining. We analyzed the organoids for morphology, DNA integrity (DAPI staining), and apoptosis with Annexin V staining/flow cytometry. First, we stained with DAPI and assessed DAPI for nuclear integrity (**Figure 6**). We did not identify significant differences in nuclear appearance between the organoids treated with individual chemotherapies and DMSO treatment. Next, we conducted staining for Annexin V to determine if there was enhanced apoptosis in UCS1 organoids treated with the monotherapies (**Figure 7**). Each individual chemotherapy agent, except ifosfamide (**Figure 7H**), had a mild increase in Annexin V staining. Of the six drugs used, gemcitabine induced the most cell death, increasing cell death from 10.539% in DMSO-treated to 34% in gemcitabine-treated cultures (**Figures 7B** vs. **7E**).

**Figure 8:**
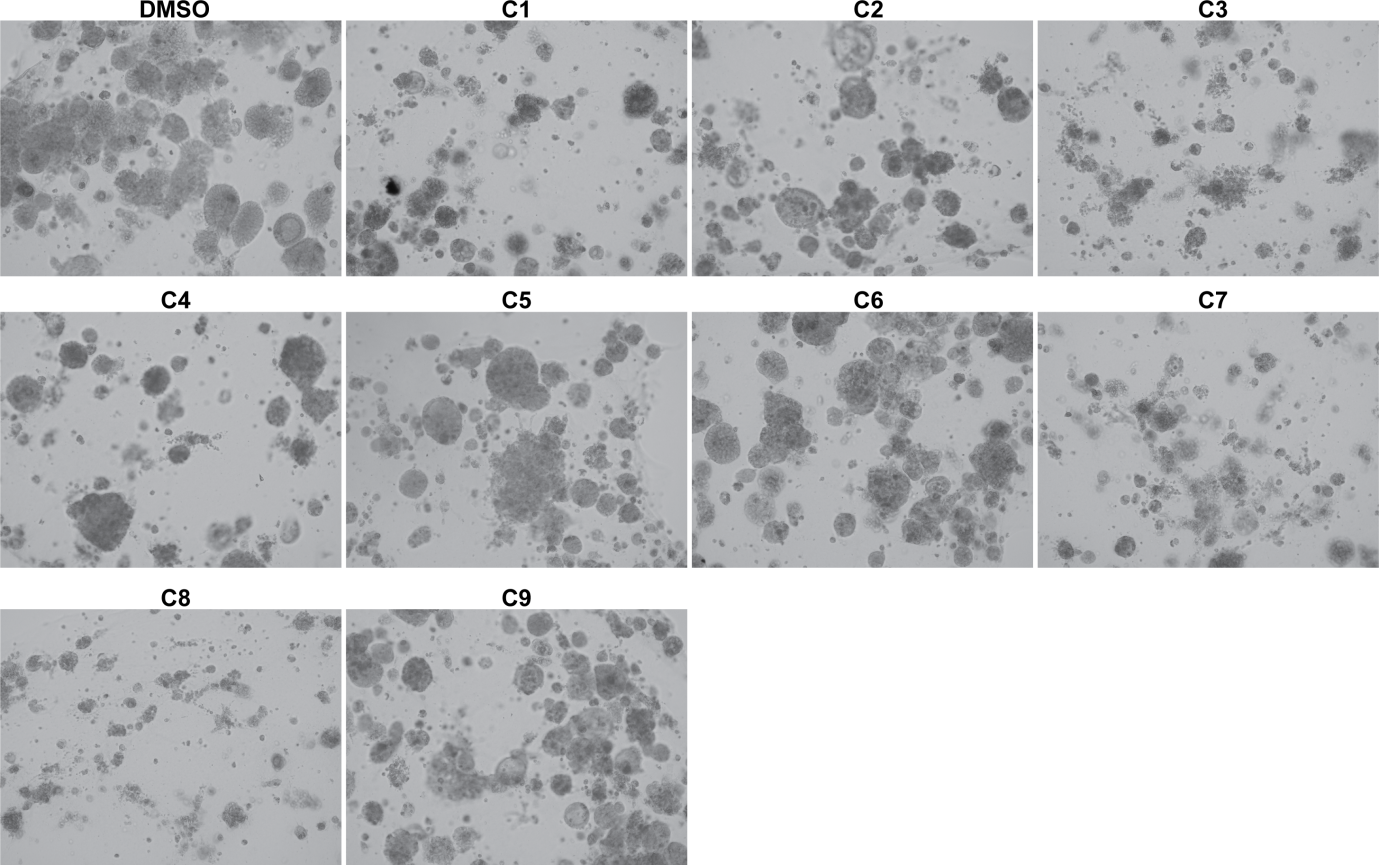
Brightfield morphology of UCS1 cells treated with combination chemotherapies. UCS1 cultures were treated with combination chemotherapy for 48 h or DMSO as a vehicle control. Brightfield images were collected to demonstrate cellular morphology. 10× magnification.

**Figure 9:**
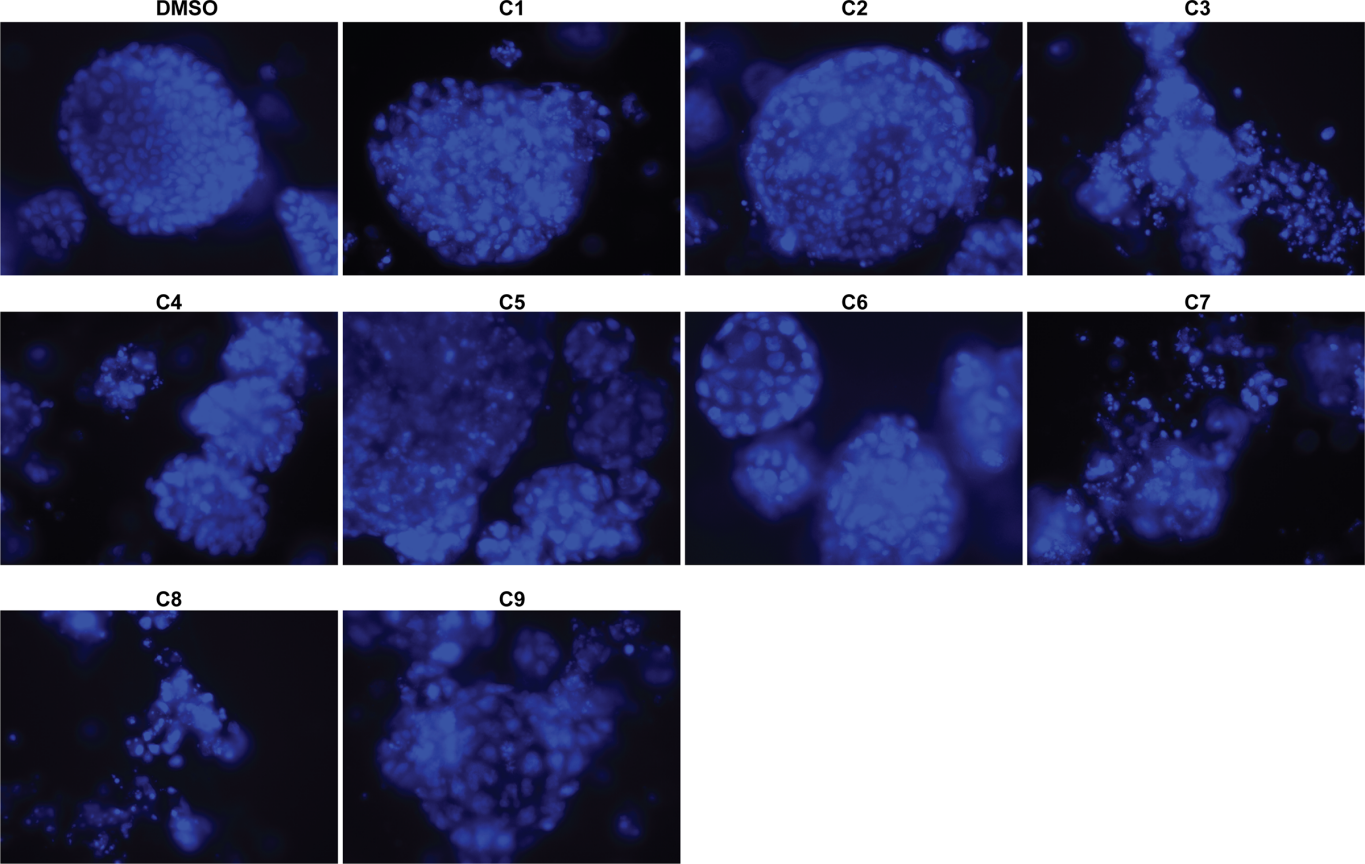
Nuclear morphology of UCS1 cells treated with combination chemotherapies. UCS1 cultures were treated with combination chemotherapy for 48 h or DMSO as a vehicle control and stained with DAPI as an indication of nuclear integrity. Fluorescent images were collected. 40× magnification.

**Figure 10:**
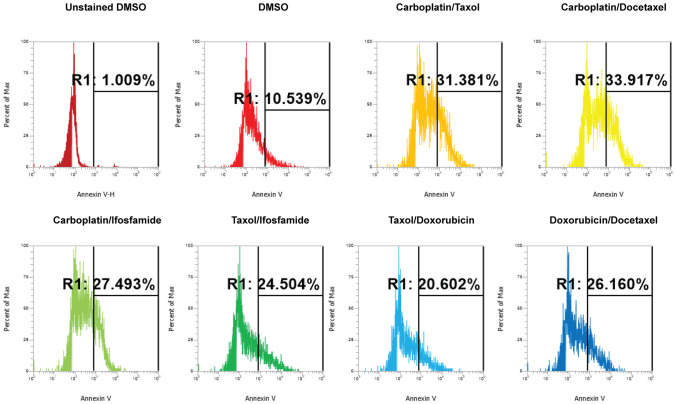
Induction of apoptosis by combination chemotherapy treatments in UCS1 PDOs. UCS1 cultures were treated with DMSO or the following combinations: C1, C2, C4, C5, C6, and C9. Annexin V staining followed by flow cytometry was conducted. Percentages of Annexin V-positive cells are indicated as compared with unstained and DMSO-treated UCS1 cultures.

**Figure 11:**
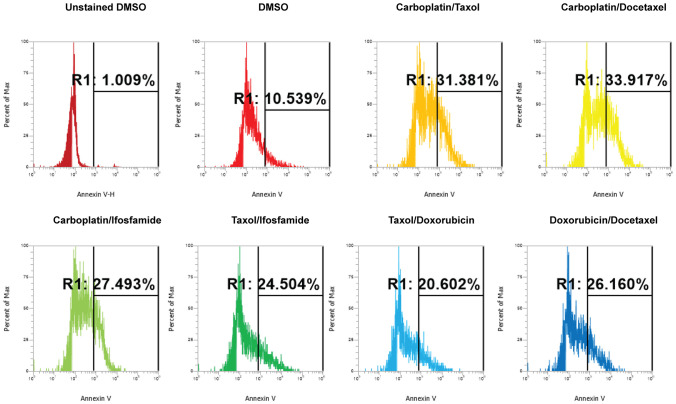
Comparison of apoptosis between UCS1 PDOs treated with carboplatin/paclitaxel (C1) or gemcitabine combinations (C3, C7, and C8). UCS1 cultures were treated with DMSO or the following combinations: C1, C3, C7, and C8. Annexin V staining followed by flow cytometry was conducted. Percentages of Annexin V-positive cells are indicated as compared with unstained and DMSO-treated UCS1 cultures.

Next, we investigated the impact of chemotherapy combinations on organoid viability. We chose to use dose 4 from **Table 1** for each drug for combination treatments (same as used for monotherapies). We compared eight combination chemotherapies (**Table 2**) to carboplatin/paclitaxel, which is the standard of care for UCS and the treatment regime that was given to the patient from which the UCS1 cell line was derived. Each of these combinations has been used to treat patients with various forms of cancer. Brightfield microscopy demonstrated compromised organoid structures for cultures treated with the C3, C7, and C8 combinations (**Figure 8**). The nuclear integrity of organoids treated with combinations 3, 7, and 8 was altered compared with treatment with carboplatin/paclitaxel and DMSO-treated cells (**Figure 9**). Nuclei in cultures 2, 7, and 8 culture were fragmented. Additionally, F-actin organization was abnormal in C3 and C7 (**Supplementary Figure 2**). Finally, we assessed by Annexin V staining the extent of apoptosis in UCS1 PDO cultures treated with the nine combinations of chemotherapy. In **Figure 10**, we compared combinations C2, C4, C5, C6, and C9 to treatment with carboplatin/paclitaxel. We stained cells with Annexin V and performed flow cytometry to determine if the different combinations of chemotherapy induced apoptosis in UCS1 tumor organoids (**Figure 10**). We found that treatment with carboplatin/paclitaxel resulted in 31% of the cells staining for Annexin V (**Figure 10**). Each combination chemotherapy increased Annexin V staining/percent of apoptotic cells compared with DMSO treatment. However, combinations C2, C4, C5, C6, and C9 were equal or inferior at inducing apoptosis compared with carboplatin/paclitaxel. In **Figure 11**, we again treated cells with either carboplatin/paclitaxel or three different gemcitabine combinations C3, C7, and C8. In this experiment, we found that the C3 and C7 combinations resulted in more cell death than carboplatin/paclitaxel. The increase in Annexin V positivity in cultures treated with the C3 and C7 combinations agreed with the fragmented DNA integrity in **Figure 9**. The combination of carboplatin/gemcitabine and paclitaxel/gemcitabine resulted in 83% and 80% of the cells labeling as Annexin V positive compared with 53.6% Annexin V-positive cells with carboplatin/paclitaxel treatment. Treatment with gemcitabine/doxorubicin resulted in 53% of the cells staining positive for Annexin V despite the abnormal appearance of the cultures and poor DNA integrity. These data suggest that the mechanism for gemcitabine/doxorubicin-induced cell death needs further investigation. Representative flow cytometry plots from four experiments were provided. Our data suggest that the UCS1 PDO exhibited increased susceptibility to gemcitabine combination therapies.

## 3 Discussion

Our goal was to generate a novel UCS organoid cell line that could be used to test currently unused combinations of chemotherapy since UCS has poor response rates to chemotherapy and radiation. We successfully generated UCS1, a PDO line that displays epithelial and mesenchymal morphologies. UCS1 was generated from a patient that had received treatment with paclitaxel and carboplatin. Our UCS1 cell line enabled us to ask if the UCS organoids still exhibited a response to the combination of carboplatin and paclitaxel or if another chemotherapy regime would exhibit an improved response. Interestingly, we found that for our UCS1 PDO line, carboplatin/gemcitabine and paclitaxel/gemcitabine were superior to carboplatin/paclitaxel at inducing cell death. The improvement in cell killing with gemcitabine combinations may have occurred for a variety of reasons that have yet to be explored. Since the UCS1 line was derived from the tumor following the patient having received carboplatin/paclitaxel, the tumor may have acquired some level of chemoresistance to carboplatin/paclitaxel from the neoadjuvant therapy. We saw minimal response to carboplatin/paclitaxel. Future studies will explore how treatment-naïve tumors respond to combination chemotherapies such as those with gemcitabine. Moving forward, it will be critical to determine if PDOs can be used to predict response to first-line chemotherapy in addition to second-line chemotherapy and beyond. We hope to achieve this by creating PDOs from treatment-naïve tumors in addition to tumor cells derived for subsequent surgeries or procedures like paracentesis. Importantly, we identified two combinations of chemotherapy that effectively induced apoptosis in UCS1. Our data suggested that organoid-derived cell lines could be used to predict second-round chemotherapy options.

Current US National Comprehensive Cancer Network (NCCN) guidelines recommend carboplatin/paclitaxel for systemic UCS therapy. Ifosfamide, ifosfamide/paclitaxel, and cisplatin/ifosfamide are also recommended regimes for UCS. In our study, ifosfamide was not effective at inducing cell death in this UCS cell line. Our results are in alignment with a recent phase III trial demonstrating that carboplatin/paclitaxel is superior (or not inferior) to ifosfamide-based regimes (29). The study further concludes that carboplatin/paclitaxel should be used to treat UCS instead of ifosfamide (29). Surprisingly, gemcitabine combinations resulted in the most cell death in the UCS1 organoids. Gemcitabine alone had a mild increase in cell death, and combining gemcitabine with carboplatin and paclitaxel further increased apoptosis. However, there are few studies on gemcitabine and UCS. Perhaps with improvements in personalized medicine, we may find that some UCS patients may benefit from gemcitabine therapy. One *in vitro* study demonstrated that gemcitabine may have a potential for use in UCS when combined with other drugs. In the UCS cell line SK-UTI-B, combining gemcitabine with mTOR inhibition resulted in additive cell death (30). Other patient studies failed to show gemcitabine as effective in UCS. In a phase II study, the combination of docetaxel and gemcitabine was not active as a second-line chemotherapy in recurrent UCS (31). It is worth noting that the gemcitabine combinations we used are effective in other tumor types. Gemcitabine combined with carboplatin increased progression-free survival in ovarian cancer patients (32). Gemcitabine/carboplatin treatment was an effective second-line treatment in metastatic breast cancer (33). Additionally, gemcitabine when combined with paclitaxel has shown effective treatment in a variety of settings including lung cancer and urothelial cancer (34–36). A recent study found that endometrial and ovarian PDOs could be generated efficiently and predicted response to therapy (28). Moreover, in one PDO for serous (not UCS) endometrial cancer, gemcitabine was superior to carboplatin/paclitaxel at killing cells. Our data further support how useful PDOs can be in determining chemotherapy sensitivity.

Currently, the chemotherapy for UCS is often carboplatin/paclitaxel. However, given the poor survival rates of UCS, there may be better treatment options that have not been explored. It is important to note that whereas our results are *in vitro* and will require *in vivo* corroboration, preclinical, and clinical studies to validate changes to current UCS treatment practices, our PDO model highlights how *ex vivo* drug sensitivity screening could be used to improve personalized approaches to chemotherapy. An additional important step in determining if gemcitabine monotherapy and combination therapies might be efficacious for some UCS patients will be to generate patient-derived xenografts from multiple patients and additionally test gemcitabine combinations in preclinical mouse models (37–39). Importantly, our data suggest that some patients may benefit from gemcitabine combinations, particularly if they have acquired resistance to carboplatin/paclitaxel. However, we do not know if many patients or only a few would benefit from gemcitabine-based therapies until more PDOs have been generated and tested. Critically, our data found that using PDOs that identify effective combinations of therapies could lead to personalized treatment for UCS.

## 4 Materials and methods

### 4.1 Generating organoids from solid tumor

Patient UCS tumor specimen was obtained at Gundersen Heath Systems for patient-derived organoid (PDO) generation. Written consent was obtained for subjects under IRB approved protocol no. 2-20-10-004. The studies were conducted in accordance with the Belmont Report and U.S. Common Rule and approved by GHS IRB. Subsequently, the pathology department provided the tissue in Advanced Dulbecco’s Modified Eagle Medium/Ham’s F-12 (DMEM/F-12) (Thermo Fisher, Waltham, MA, USA). The medium was supplemented with 50 U/ml penicillin, 50 U/ml streptomycin, 1% GlutaMAX, and 10 mM HEPES. Cell culture additives were purchased from Thermo Fisher (Waltham, MA, USA). Tumor organoids were generated from tumor as described (40). Briefly, a single-cell suspension of tumor cells was generated by dicing tumor into small pieces and digesting with collagenase. Cells were then ACK lysed to remove erythrocytes, and cells were counted. Cells were resuspended in artificial Basement Membrane (Cultrex 3D Culture Matrix Reduced Growth Factor Basement Membrane Extract) (Bio-Techne, Minneapolis, MN, USA) and plated in domes on 48-well plates. Basement Membrane domes were incubated at 37°C for 5 min. Once the Basement Membrane solidified, defined media described in (40) were added to each well. Media on the organoids were changed every 3–4 days for maintenance.

### 4.2 Chemotherapy treatments

Organoids were collected from Basement Membrane by incubating organoids in TrypLE Express (Thermo Fisher, Waltham, MA, USA) followed by an incubation at 37°C for 5 min. Organoids were mechanically dislodged from the culture surface *via* pipetting up and down, incubated on ice for 1 h, and spun at 1,200 RPM for 10 min at 4°C. After supernatant was removed, cells were resuspended in Basement Membrane. Domes were plated on individual wells of 48-well plates at 10,000 cells per dome. Once gel polymerizes, media were added to each well. Organoids grew for 1 week before receiving chemotherapy treatments. At time of treatment, media were removed from the domes and replaced with media containing drugs as listed in **Tables 1**, 2.

### 4.3 Immunofluorescence

Organoids in Basement Membrane domes were plated on four-well chamber slides. Cells were fixed in warm 3.7% formaldehyde in PBS for 15 min at 37°C. Cells were permeabilized and blocked with bovine serum albumin (BSA) in Tris-buffered saline with 0.1% Tween (TBST). UCS1 organoids were incubated with primary antibodies and/or Texas Red phalloidin (Invitrogen, Carlsbad, CA) for 1 h at 37°C. Cells were washed with TBST thrice. For PAX8 and p53, cells were incubated with conjugated secondary antibodies for 1 h at 37°C followed by rinsing with TBST. Fluoromount-G with DAPI (Thermo Fisher, Waltham, MA, USA). Images were collected using an EVOS M5000 (Invitrogen, Carlsbad, CA) or the Leica STELLARIS 5 laser confocal microscope (Leica Microsystems, Inc., Deerfield, IL). IF was evaluated by KDCD and KAL.

### 4.4 Pathology

The Pathology Department at Gundersen Health Systems used standard H&E and immunohistochemistry (IHC) techniques were used to stain for p53 and cytokeratin AE1AE3 on the UCS patient tumor.

### 4.5 Annexin V flow cytometry

Organoids were collected from Basement Membrane by incubating organoids in TrypLE followed by an incubation at 37°C for 5 min. Organoids were mechanically dislodged from the culture surface *via* pipetting up and down, incubated on ice for 1 h, and then spun at 1,200 RPM for 10 min at 4°C. Cells were washed with PBS and Annexin V Binding Buffer (10 mM HEPES, 140 mM NaCl, 2.5 mM CaCl_2_). Then, cells were incubated in Annexin V Binding Buffer with Annexin V-PE (BD Biosciences, Franklin Lakes, NJ) for 15 min at room temperature. Cells were washed with Annexin Binding Buffer. Finally, cells were spun down and resuspended in 0.1% BSA in PBS. Flow cytometry was performed on an Attune NTX flow cytometer (Thermo Fisher, Waltham, MA, USA).

### 46 Dose curve

To identify the optimal doses for each of the six chemotherapies that we used, we conducted a dose curve using the concentrations in **Table 1**. Briefly, we plated organoids (10,000 cells per dome) in Basement Membrane domes on individual wells of 48-well plates. Once gel polymerizes, media were added to each well. Organoids were grown for 1 week before receiving chemotherapy treatments. Each treatment was performed in triplicate. To measure cell death, we stained cells with CellEvent Caspase3/7 (Thermo Fisher, Waltham, MA, USA) according to instructions. Briefly, we added CellEvent Caspase3/7 to each culture for 30 min at 37°C along with NucBlue (Thermo Fisher, Waltham, MA, USA) to denote nuclei. We visualized dead cells (apoptotic) using fluorescence microscopy. We took images of each condition and scored cultures for the intensity of fluorescence. A score of 0 was no fluorescence and 3 was the highest intensity.

### 4.7 Chemicals

Texas Red phalloidin and Fluoromount-G with DAPI were purchased from Thermo Fisher (Waltham, MA, USA). Carboplatin, paclitaxel, gemcitabine, doxorubicin, docetaxel, and ifosfamide were all purchased from (Santa Cruz Biotechnology) (Dallas, TX, USA). Dimethyl Sulfoxide (DMSO) was purchased from Sigma-Aldrich (St. Louis, MO, USA).

### 4.8 Antibodies

The antibodies for Cytokeratin AE1AE3 (AE1/AE-601-L-CE) was purchased from Leica Biosystems (Deer Park, IL, USA), and p53 antibodies (Clone D0-7) are from (Dako/Agilent, Santa Clara, CA, USA). Anti-PAX8 and anti-Vimentin antibodies were purchased from Santa Cruz Biotechnologies (Dallas, TX, USA). We obtained antibodies against E-cadherin and N-Cadherin from BioLegend (San Diego, CA, USA).

## Data availability statement

The original contributions presented in the study are included in the article/**Supplementary Material**, further inquiries can be directed to the corresponding author/s.

## Ethics statement

The studies involving humans were approved by Gundersen Health Systems Institutional Review Board. The studies were conducted in accordance with the local legislation and institutional requirements. The participants provided their written informed consent to participate in this study.

## Author contributions

MD: Investigation, Writing – original draft. KL: Data curation, Writing – review & editing. CH: Investigation, Writing – original draft. GH: Resources, Writing – review & editing. SH: Formal analysis, Resources, Writing – review & editing. KC: Conceptualization, Formal analysis, Investigation, Project administration, Writing – original draft, Writing – review & editing.

## Supplementary material

Supplementary Figure 1 Dose curve on the six chemotherapies applied to UCS1 organoids. UCS1 organoids were treated with DMSO (Dose 1) or increasing concentrations of carboplatin, docetaxel, doxorubicin, gemcitabine, ifosfomide, or paclitaxel (See ). Cells were stained with CellEvent Caspase 3/7 and intensity of caspase activation/fluorescence was scored on a scale of 0 (no fluorescence) to 3 most intense fluorescence. DMSO treatment was 0 (not shown on graph) as was Dose 2 of carboplatin.

Supplementary Figure 2 Nuclear and cellular morphology of UCS1 cells treated with gemcitabine combination chemotherapies. UCS1 cultures were treated with DMSO, Carboplatin/Paclitaxel (C1), Carboplatin/Gemcitabine (C3) or Gemcitabine/Paclitaxel (C7) for 48 hours or DMSO DAPI as an indication of nuclear integrity or Texas-Red Phalloidin. Fluorescent images were collected. 10x magnification.
