# Supplementary figures and images for: Gemcitabine combination therapies induce apoptosis in uterine carcinosarcoma patient-derived organoids
Source: Front Oncol. 2024 Mar 13;14:1368592. doi: 10.3389/fonc.2024.1368592 (PMC10966467; doi:10.3389/fonc.2024.1368592)

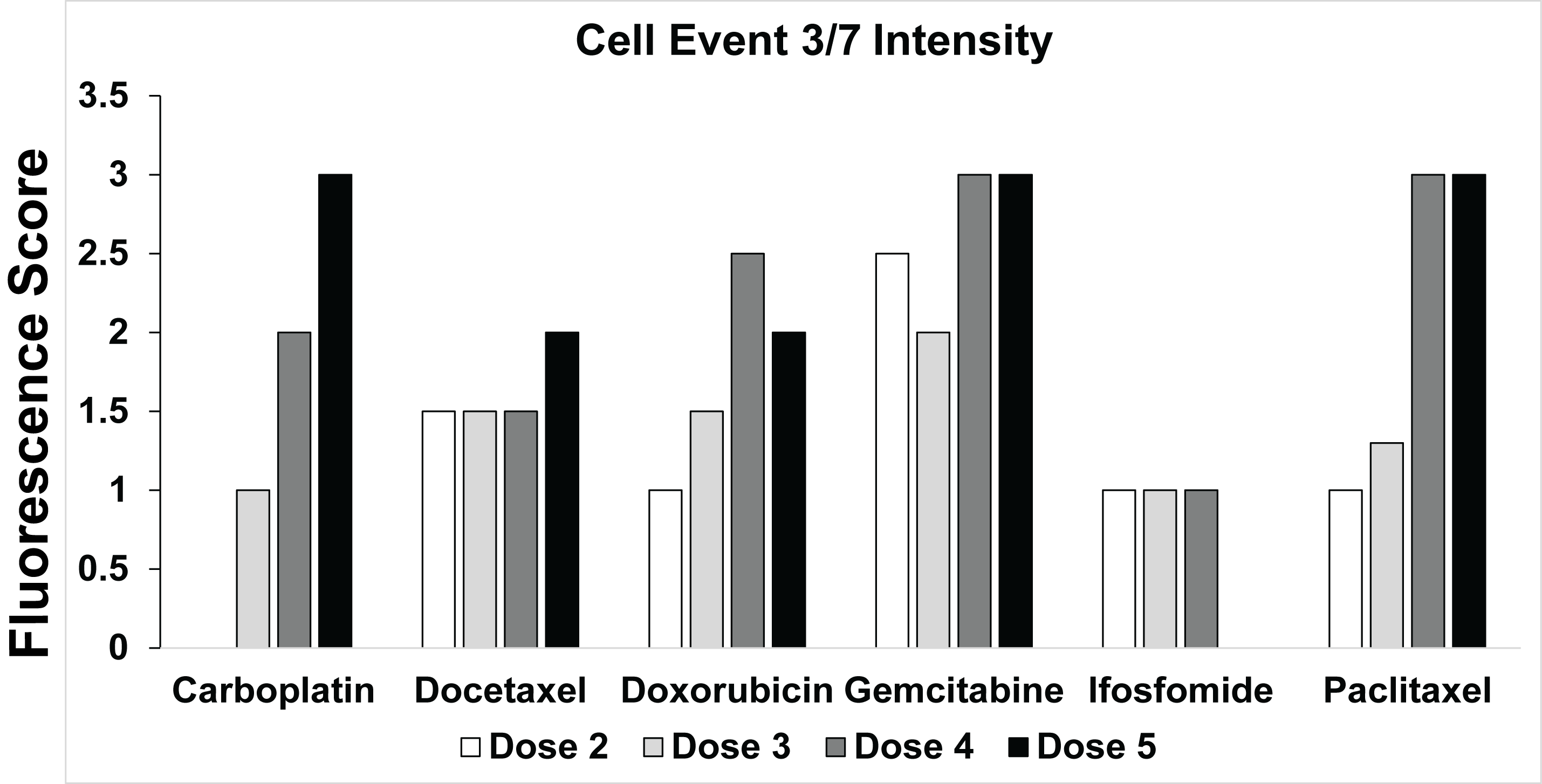

Supplement: Supplementary Figure 1 — Dose curve on the six chemotherapies applied to UCS1 organoids. UCS1 organoids were treated with DMSO (Dose 1) or increasing concentrations of carboplatin, docetaxel, doxorubicin, gemcitabine, ifosfomide, or paclitaxel (See ). Cells were stained with CellEvent Caspase 3/7 and intensity of caspase activation/fluorescence was scored on a scale of 0 (no fluorescence) to 3 most intense fluorescence. DMSO treatment was 0 (not shown on graph) as was Dose 2 of carboplatin. [file Image_1.tif]

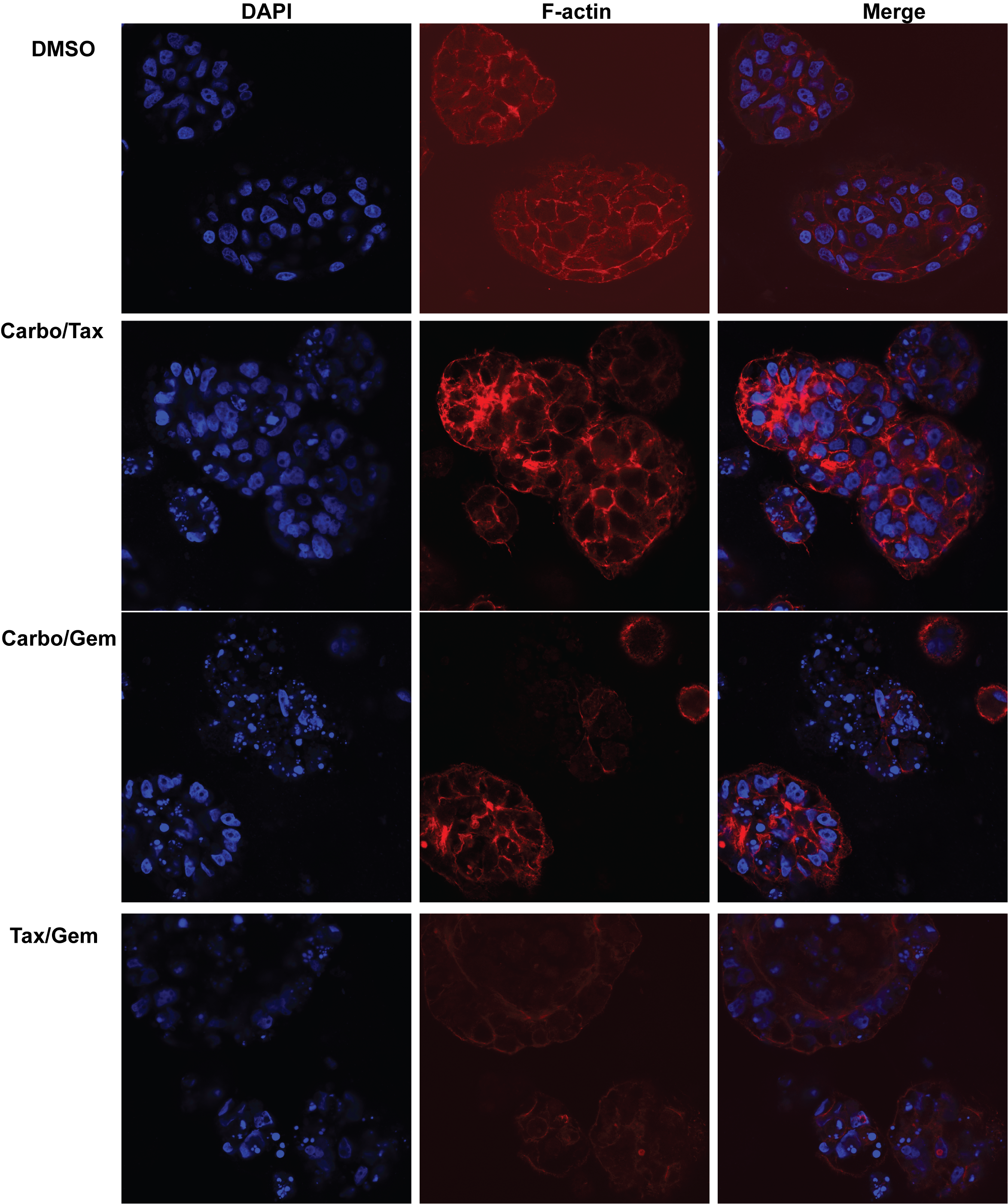

Supplement: Supplementary Figure 2 — Nuclear and cellular morphology of UCS1 cells treated with gemcitabine combination chemotherapies. UCS1 cultures were treated with DMSO, Carboplatin/Paclitaxel (C1), Carboplatin/Gemcitabine (C3) or Gemcitabine/Paclitaxel (C7) for 48 hours or DMSO DAPI as an indication of nuclear integrity or Texas-Red Phalloidin. Fluorescent images were collected. 10x magnification. [file Image_2.tif]
